# Concluding Remarks for the Special Issue on RNA Viruses and Antibody Response, Second Edition

**DOI:** 10.3390/v16111678

**Published:** 2024-10-28

**Authors:** Heng Yu, Jianning He, Yiu-Wing Kam

**Affiliations:** Division of Natural and Applied Science, Duke Kunshan University, No. 8 Duke Avenue, Kunshan 215316, China; heng.yu@dukekunshan.edu.cn (H.Y.); jianning.he@dukekunshan.edu.cn (J.H.)

The emergence and re-emergence of RNA viruses pose a constant, significant global health problem [1]. The potential threat posed by RNA viruses to human society has persisted into the post-pandemic era, and this grim reality requires an understanding of the epidemiology, pathogenesis, and treatment of these viruses. Furthermore, one can learn from previous outbreaks to improve disease control and minimize the health threat of RNA viruses and their enormous impact on normal socio-economic order [2]. Disease control measures include, but are not limited to, the mathematical modeling of infectious diseases, the development of novel laboratory case detection methods, vaccine development, and novel vaccination delivery strategy. The neutralization of antibody responses plays a crucial role in the construction of all these approaches, and clarifying the kinetics of antibody–virus interactions will better enhance the effectiveness of these measures [3]. This Special Issue will help readers gain a deeper understanding of valuable knowledge and findings related to antibodies.

“RNA Viruses and Antibody Responses” is now in its second edition. In the first edition, 14 original studies and 2 reviews were published by researchers from around the globe, including 11 original papers related to SARS-CoV-2, with the remaining studies and reviews dealing with the mechanisms of infection and antibody knowledge of other RNA viruses (e.g., influenza viruses, chikungunya virus (CHIKV)). In the second edition, seven original studies and three reviews were published by researchers globally, including three original papers related to SARS-CoV-2, with the remaining studies and reviews focusing on other RNA viruses (e.g., human immunodeficiency virus (HIV), Andes Orth hantavirus (ANDV), infectious bronchitis virus (IBV), dengue virus (DENV), etc.). As shown in Figure 1A, in the second edition, although some contributing scholars are still very interested in SARS-CoV-2 research, academics seem to be shifting their focus to issues that have plagued the field of RNA virus research for many years, such as HIV and DENV. This shift and expansion of the research basis will help to refocus attention on previous outbreaks of RNA virus-related diseases in the post-pandemic era and on how to better manage and control the spread of RNA virus diseases other than coronaviruses through new research discoveries. In addition, influenza virus research has also been reported in both Special Issues. Influenza viruses are a significant public health concern, causing seasonal epidemics and occasional pandemics with potentially severe consequences. It is important to highlight more research and academic discussions about influenza viruses, as these are vital for protecting public health against influenza-related morbidity and mortality.

Meanwhile, as shown in Figure 1B, the articles in this Special Issue mainly focus on cohort studies, although there are also a few review articles, which can help to deepen the general knowledge of RNA viruses, in addition to a general summary of some RNA viruses, and some specific studies on antibody response. We also note that the construction of mathematical models of infectious diseases is a common concern in both Special Issues. Also, unlike the first Special Issue, which focused on the diagnosis and treatment of RNA virus diseases, the second Special Issue has shifted its focus to the development of vaccines; this can be regarded as a result of the advances made in our knowledge of disease prevention and control.

The second edition collects together two original research papers and one review related to SARS-CoV-2, both discussing antibody kinetics. The two studies discuss the correlation of novel chimeric recombinant antigens and clinical features with antibodies, respectively, and provide important information for the development of more reliable laboratory assays and for the selection of suitable neutralizing antibody-containing plasma donors. The related review examines the impact of antiglycan antibodies on the triggering of the antibody-dependent enhancement (ADE) phenomenon.

This Special Issue also contains two original research papers focusing on mathematical modeling and epitope mapping for the in-depth study of antibody dynamics in RNA viruses, the application of which could contribute to future disease control and vaccine development. In addition, this Special Issue contains three studies and two reviews that explore our knowledge of antibodies associated with RNA virus infections. We may gain knowledge from the epidemiology and virology of viruses such as avian influenza and Bunyaviruses to help us structure our response to future outbreaks.

We would like to thank all the authors for their innovative and thoughtful research and review articles in this Special Issue, which will inspire the academic community to continue their efforts in the field of RNA virus and antibody research and provide new ideas or insights for researchers in the same field. We hope that this Special Issue will continue to serve as a vehicle for encouraging research in the field of RNA viruses, promoting the prevention, control, diagnosis, and treatment of RNA viral diseases and improving the ability of society as a whole to cope with future epidemics.

## Figures and Tables

**Figure 1:**
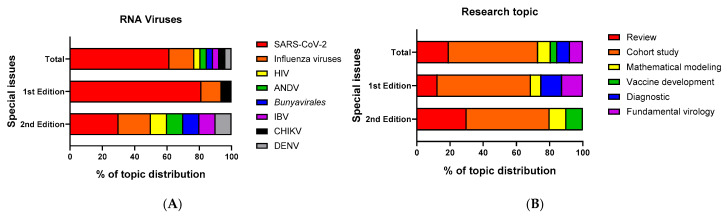
The percentage distribution of the topics covered in the Special Issue “RNA Viruses and Antibody Response”. (**A**) Bar chart of the percentage of papers discussing viruses in the 1st (August 2022–April 2023) and 2nd (June 2023–July 2024) editions of this Special Issue. The bars are divided into three sections, Total, 1st edition, and 2nd edition, representing two editions of the Special Issue “RNA Viruses and Antibody Response”. Each color within the bars represents a different virus (either a specific species of RNA virus or a group of viruses in the same family of RNA viruses), and the length of each segment corresponds to the percentage of papers dedicated to that virus. (**B**) Bar chart of the percentage of papers covering a specific research topic in the 1st and 2nd editions of this Special Issue. Each color within the bars represents a particular research topic, and the length of each segment corresponds to the percentage of papers dedicated to that research topic.
